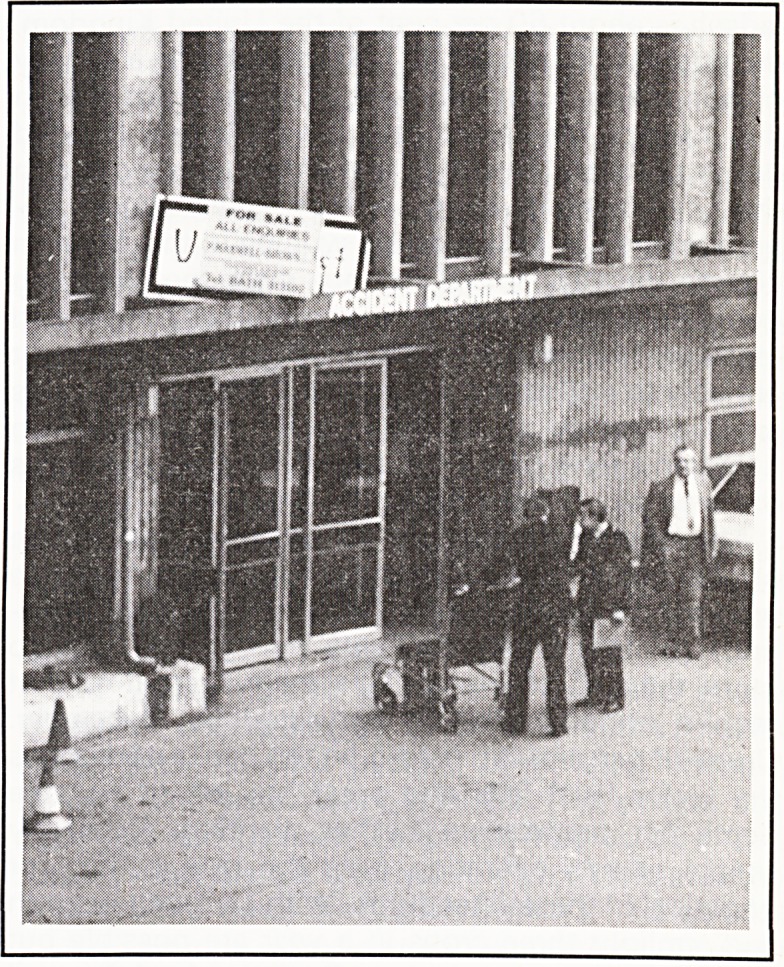# From Our Correspondents

**Published:** 1985-04

**Authors:** 


### From Our Correspondents

### Our Image

During a recent discussion on one of the fashionable ethical problems of our time, a lawyer colleague said rather vehemently that 'he didn't see why society should be dictated to by doctors'. I refrained from the immediate rejoinder which came to my mind about that being infinitely preferable to being dictated to by lawyers, because I did not think it would be helpful, and am not so certain that it is true. What I did do, however, was to remind him that doctors were, in fact, *part of* society rather than outsiders looking in. I mention this only to make the point that the general perception of us today is frequently other than the caring, concerned profession we consider ourselves to be. Sadly, even (or perhaps particularly) in the non-medical hierarchy of the Health Service consultants are too often categorised as self-serving, high-technology besotted, liabilities who are for ever neglecting their NHS responsibilities for private practice and, as a result can never be found when required. Nor do general practitioners fare any better. Their laziness is legendary, is it not? Why else should they rely so heavily on deputising services, and isn't it a 'well known fact' that only 20% of their time is spent seeing patients?

Now, every one of us knows one or two of our colleagues who approximate to a greater or lesser extent to these caricatures (Don't worry, Bill, your secret is safe with me!) but does either of the descriptions do any justice to how you perceive your role? Of course not – indeed, in my view, cool assessment of verifiable facts would place the truth nearer the totally opposite view to that expressed above. But why are our public relations so bad and our public persona so different from what we perceive to be true? One clue lies within the statements which usually accompany the invective. 'We must bring them to book,' they say; 'they must be made more accountable'. Thus it seems it is our cherished professional freedom which appears to pose such a threat. But this merely causes our puzzlement to deepen – aren't we more hedged about by controls and bureaucracy and therefore *less* free or independent in the NHS than we ever have been before? Doesn't every re-organisation (including the latest and most profound – the Griffiths proposals!) distance us even more than the last from the front line of patient care which is our primary responsibility? Be that as it may, we must be at least responsible for our poor image and for the benefit of everyone, particularly those for whom we strive earnestly to care, it would be as well for us to try to redress the balance as soon as possible. To do so we must firstly try to understand why it has occurred; secondly, refute it with facts at every opportunity; and thirdly, be more willing to discipline those few members of our profession whose misdemeanours affect us all.

G. M. Stirrat

### Desert Island Drugs

I sometimes ask my students to write down the twenty drugs which they consider essential for survival on a desert island. It is remarkable how this exercise concentrates the mind on the really important medications. Most of us who have served in the armed services or worked in the third world know perfectly well that we can practise good medicine and surgery with very limited facilities, drugs and instruments.

I was, therefore, ashamed of the emotional response by the B.M.A. and some of our colleagues to the D.H.S.S.'s proposed limitations on prescribing published in November. None of the restrictions were for life-saving drugs such as antibiotics, insulins, antimalarials, and the list retained two of my desert island drugs – aspirin and aluminium hydroxide. There were just a few omissions which needed to be re-instated, and unfortunately the mode of presentation was rather abrupt. However, as a result of intensive lobbying by some doctors, and liberal funds and fuel from the drug industry, Mr. Fowler has yielded to pressure and allowed three times the number of preparations. There surely is no pharmacological reason for us needing:—

(a) Nine different bulk laxatives when altering dietary habits is usually as effective.

(b) Thirty-five different formulations of mild analgesics.

(c) Five different strengths of ascorbic acid, in a country where fresh fruit and vegetables are available all the year round.

(d) Four preparations of Vitamin E when there is no scientific evidence of any benefit.

(e) Ammonium chloride mixture and simple linctus as "expectorants." Licquorice allsorts and home made lemon and honey are more palatable and effective.

The unfortunate effect of this increased list is that there will be £25 million less saved. That means less money available for increasing the facilities for hip and heart operations, kidney dialysis, care of the terminally ill, home nursing, etc. One can only hope that the profession will now prescribe responsibly and economically on a voluntary basis using a limited number of preparations, if for no other reason than cutting down the number of preparations that a pharmacy has to stock. As a practising retail pharmacist told me this week, he has a tremendous waste of medicines through having to store so many preparations with similar activity.

H. G. Mather

**Ham Green Hospital**

The Ham Green Estate containing 99 acres of land and including a mansion, two farms, five cottages and outbuildings was sold to the Corporation of Bristol in 1893 for £8,695. Thirty eight acres was allocated to hospital grounds. New buildings were created on the site, materials being conveyed by road and water, and also unloaded at a Railway Station (Pill) three quarters of a mile from the site and then having to be hauled up a very steep hill.

The hospital, with 76 beds was officially opened on Wednesday 12 July 1899. Eventually, with a road running through the middle, beds on one side were used for patients with infectious diseases and on the other side for tuberculosis. The patients were housed in a number of separate buildings to avoid cross infection. The number of beds steadily increased until there were over 400 at one time. Fortunately, no major damage occurred to the hospital during the two wars.

Visiting by relatives was not allowed in the early days of the hospital, one only learnt how ill the patients were by reading the local paper where they were listed by a number.

Right from the start the hospital realised that in order to look after the patients one should look after the staff and Ham Green was one of the first in the country to develop an Occupational health service. This started with immunisation and TB testing and later extended to provide a full service for all staff.

The introduction of Penicillin later in the 1939–45 war was a major step forward in the treatment of infection. This was quickly followed by other antibiotics including Streptomycin, which was life saving in the hitherto fatal tuberculous meningitis.

In the 1950's new techniques were developed in artificial respiration to treat firstly poliomyelitis (prior to this there had only been the tank respirator (iron lung) and later tetanus – the severe cases requiring tracheotomy and Positive Pressure Respiration. Now, with immunisation, tetanus has become fortunately rare in the UK and cases which are seen are usually treated by anaesthetists in intensive care units.

In December 1959 the hospital became a mainly acute hospital – administered by the Southmead Management Committee, having previously had its own management. It was a little later (in the 1960's) that the hospital was among many first threatened with closure, but at that time the need to keep open was established and further specialties acquired, the hospital always having reponded to the challenge of change and the needs of the Community. Over the years additional units included a purpose built one for the chronically disabled, a Clinical Investigation Unit for the study and treatment of urinary incontinence, in patient and day care for elderly and also elderly mentally infirm. As the need for beds for TB declined new uses were found for many of the beds including gynaecology and general surgery with eventually new theatre facilities providing a service for patients from the Weston area.

In the 1960's the hospital was the first in the area to use haemodialysis for the treatment of renal failure and this continued for many years until the purpose built unit was opened at Southmead.

In the 1970's a Coronary Care Unit was created within a general medical ward and for many years considerable sums of money have been raised by voluntary organisations, including our own League of Friends, to buy both equipment and to furnish many areas in the hospital. Also during this period a special unit was built to handle exotic diseases including Lassa fever.

Changing needs are again recommending closure of the hospital, which is one of many scheduled for closure in districts in the Area 10 year Strategic Plans. Other possible uses for at least part of the hospital complex are being considered before a final decision is made. Meanwhile, the hospital will continue to serve the Community as it has done during the past 85 years.

This report has, by necessity, only been able to give a general picture of some of the work of the hospital over the years since it was opened and no mention has been made of the many staff, doctors, nurses and others, who have been responsible for making it both well known and respected, and for creating a happy atmosphere for the staff to work in providing care for their patients.

D. W. Wright

**The Cossham Medical Society**

In 1946 the general practitioners practising in the district around Cossham Hospital decided to form a learned Medical Society. Many of them had just returned from the war, the remainder had minded their absent neighbours practice's during the conflict. At that time there was no post-graduate teaching other than a formal professorial Sunday morning round in the B.R.I. The concept of a learned Society was both novel and way ahead of the times. 48 G.P.'s attended the inaugural meeting when Dr. P. T. McDonald from Kingswood was elected the first President. It was agreed that, on the first Tuesday of every winter month a distinguished expert should be invited to address the members on his subject, hopefully to the benefit of the listeners; it was also decided that membership subscriptions should be half a guinea, and that there should be a cocktail party on the last Saturday in November, (of which more anon).

The inaugural lecture was given by Professor Bruce Perry entitled 'The Application of Penicillin Therapy'. The meetings were originally held in the library of Cossham Hospital; if an above average attendance was anticipated, the mortuary room in the basement of the hospital was cleared. The first meeting that I attended was held in this room, and Harry Sheppard filled and entertained all in the morgue with anecdotes concerning his honorary post as Bristol Zoo obstetrician (a vaginal exmination of a labouring giraffe was complicated by his finger being bitten by the foetus). By 1960 membership had grown to 74 and the first consultant was proposed as a member. Is it significant that he was Dr. R. E. Hemphill, the distinguished psychiatrist?

It is not clear what the founding members had in mind for a cocktail party. It is apparent from early financial reports that the profits in the early years were so great that the surplus could subsidise a social event. However, the tradition was quickly established of gathering in the President's home (the tenure of the President is one year); tongues were loosened by Barty Orten's champagne cocktails (or to be precise 'whore's blushes'), eating the supper brought by the wives of the committee, drinking the wine selected by the President, and finally rolling back the carpets for dancing until the small hours. There have never been any tickets or advance knowledge how many would come, invariably 70–75 turn up (never the same 75).

With the opening of the Frenchay Post-Graduate Centre and the explosion of post-graduate education in the early 70's, the Society, somewhat reluctantly, moved to the Post-Graduate Centre, where we have been made most welcome.

The first consultant President was Herbert Bourns in 1974. The second, Colin Davidson in 1977 had the foresight to broaden the base of the meetings and have some open evenings for spouses and non-medical visitors. Latterly Chris Burns-Cox decreed that every meeting shall be open. Recent titles vary from "18th Century Glass" to Perinatology in the Bible". The lectures are invariably entertaining and well attended.

The 25th Anniversary was celebrated with the planting of a flowering cherry outside the Post-Graduate Centre by the oldest surviving President Dr. S. F. Marwood. We have not yet decided on the appropriate celebration in 1986. Dr. Ian McDonald, the son of the President and the only surviving founder member, is giving the matter some thought. We trust that he will have many ideas for the 40th celebration of this unique Medical Society which is of such good value, the subscription having risen from half a guinea to two pounds in 38 years. Alas the cocktail party can no longer be subsidised!!

Bernard Whiteside

**Who wants hospitals?**

We have all heard stories about the wealthy client who goes to a decaying shop or hotel. He is either dissatisfied or just plain restless, and instead of making a simple purchase or merely staying overnight, he decides to buy the entire concern on the spot.

Recently on a week-end visit I heard a variant of this type of tale. A millionaire's second daughter was expelled from an exclusive girls' school in the Home Counties. The father's response was not yours or mine. No wrath: pure cool. He simply bought the school the very next week.

How long will it be before this happens to one of our hospitals? The elements are there. Financial instability. Services at full stretch. The details of the scenario are not difficult to imagine. You can't say that we haven't been warned. How soon will it take? It is already a feature of the central London property market.[1] Who wants an N.H.S. hospital?

Jack Davies

**REFERENCES**

1. Huntley, J. and Warman, C. (1985) For Sale. *The Times* 15 January, issue 62036, p. 19.

## Figures and Tables

**Figure f1:**